# Trajectories of posttraumatic stress disorder (PTSD) and posttraumatic growth among victims 6 months after the 2021 Henan floods: predictive roles of social support and short video exposure during the disaster

**DOI:** 10.1186/s40359-025-03807-5

**Published:** 2025-12-20

**Authors:** Yimei Zhang, Junhua Dang, Zhihao Ma

**Affiliations:** 1 Computational Communication Collaboratory, School of Journalism and Communication, Nanjing University, Nanjing, 210023 China; 2 Faculty of Humanities and Social Sciences, Xi’an Jiaotong University, Xi’an, China; 3 Department of Surgical Sciences, Uppsala University, Uppsala, Sweden; 4 Provincial Key Laboratory of Intelligent Communication and Digital Society Governance, Shenzhen University, Shenzhen, China

**Keywords:** Posttraumatic stress disorder (PTSD) symptoms, Posttraumatic growth (PTG), Social support, Short video exposure, Latent growth curve model, 2021 henan floods

## Abstract

**Background:**

While cross-sectional studies have established that social support and media exposure are associated with PTSD symptoms and Posttraumatic Growth (PTG), the longitudinal dynamics of these relationships in the context of digital-era disasters remain underexplored. In particular, the psychological impact of short video exposure about the floods during disasters is not well understood. This study seeks to elucidate the predictive roles of social support and short video exposure during the 2021 Henan floods on the initial levels and changes of PTSD symptoms and PTG six months post-disaster.

**Methods:**

We conducted the study from July 20, 2021, to January 30, 2022, involving individuals affected by the disaster. A total of 279 disaster victims completed self-reported PTSD symptoms and PTG assessments at three time points (0-, 3-, and 6-months post-disaster). Utilizing a latent growth curve model, we investigated the influence of social support and short video exposure during the disaster on the initial levels and changes in PTSD symptoms and PTG.

**Results:**

Findings indicated no significant relationship between social support and PTSD symptoms. However, social support during the disaster exhibited a positive association with the initial levels of PTG, predicting lower rates of change in PTG over time. Participants with more frequent flood-related short video exposure during the disaster reported higher initial levels of PTSD symptoms, followed by lower rates of change over time. Furthermore, short video exposure positively affected the initial levels of PTG but had no impact on changes in PTG.

**Conclusions:**

This study provides preliminary evidence of the distinct roles of social support and short video exposure in relation to PTSD symptoms and PTG trajectories following a disaster, which suggests that longitudinal approaches are important for understanding the evolving impact of both social and media-related factors.

## Background

Natural disasters, which have potentially devastating effects, not only result in the loss of material resources, but also directly threaten victims’ lives and cause short and long-term psychological consequences [1]. Disaster victims may experience a wide range of mental health problems, such as depression, anxiety, and substance abuse [2–4]. Posttraumatic stress disorder (PTSD) is the most commonly studied post-disaster psychopathology [5], and PTSD symptoms are significant in a proportion of individuals following traumatic events [6]. Although the majority of psychological research on disaster victims only points to the negative effects of trauma exposure, studies have also found the positive changes facilitated by disasters. A concept related to this phenomenon is posttraumatic growth (PTG), which is associated with five different aspects: relating to others, new possibilities, personal strength, spiritual change, and appreciation of life [7, 8].

Numerous studies have investigated the factors associated with PTSD symptoms and PTG in the context of natural disasters. Research has identified social support as a protective predictor of PTSD symptoms [9]. According to the model of PTG [7, 8], social support can promote PTG through the cognitive processing of trauma [10]. Some studies have also demonstrated the beneficial role of social support in PTG [11, 12]. Additionally, media exposure has been shown to amplify perceived risk and contribute to downstream psychological effects, as evidenced by empirical studies of disaster reporting and public responses [13, 14]. Meanwhile, due to differences in personal traits and media content, exposure to media may be beneficial for posttraumatic recovery and fostering PTG in some populations because it can facilitate deliberate rumination [15].

While the cross-sectional relationships between social support and PTSD symptoms and PTG, and media exposure and PTSD symptoms and PTG have already been established, it is crucial to uncover the longitudinal relationships that contribute to a better understanding of the trajectories of psychological outcomes among disaster victims. Furthermore, with the fast development of the media ecology, the influence of emerging media on mental health, especially short video platforms, is still underestimated [16]. Therefore, to fill these research gaps, the present study uses the Latent Growth Curve Model (LGCM) to examine how social support and short video exposure during the disaster predict the trajectories of PTSD symptoms and PTG during and after the disaster, using data collected among individuals who experienced disaster exposure of the 2021 Henan floods. From July 17 to early August 2021, devastating floods hit Henan Province, China [17], leaving 302 dead and 50 missing by August 2021 [18]. It’s important to elaborate the trajectories of PTSD symptoms and PTG among those affected by the disaster. The current study may also present an opportunity to understand the influence of social support and short video exposure on the development of PTSD symptoms and PTG during disasters.

### The relationship between social support and PTSD symptoms and PTG: social causation and stress-buffering perspectives

Guided by the social causation model and the stress-buffering hypothesis [19, 20], we conceptualize perceived social support during the disaster as a proximal protective factor that can shape both baseline severity and short-term change in posttraumatic outcomes. The social causation model posits that social conditions and resources exert causal influences on mental health [19, 21, 22], whereas the stress-buffering account specifies how supportive ties attenuate the impact of stressors on symptom trajectories [20, 23, 24].

Previous cross-sectional studies suggest that disaster survivors reporting lower levels of social support are significantly related to more severe symptoms of PTSD [25, 26]. However, cross-sectional designs limit the ability to examine causal relationships. Longitudinal designs provide an opportunity to demonstrate the complex relationship between social support and PTSD symptoms. For instance, a study reported that the social causation model, which states that more social support leads to less PTSD symptoms, accounts for the support-to-stress relationship in the early post-disaster period [19]. Moreover, recent studies have started to adopt a more nuanced approach by investigating the trajectories of PTSD symptoms during or after disasters, indicating that the development of PTSD symptoms can be predicted by various factors including trauma exposure [27], relationship status [28], locus of control, and coping styles [29]. To date, however, few studies have focused on the predictive role of social support in the trajectories of PTSD symptoms.

Both social causation model and stress-buffering hypothesis propose that social support as a resource empowers individuals to cope with crisis and then contributes to the posttraumatic psychological recovery [30–33]. Social support has been identified as a predictor of PTG. The positive association has been demonstrated in individuals who have experienced a variety of traumatic events, such as burn patients [34] and cancer survivors [35]. However, inconsistent findings exist in cross-sectional designs, with research revealing non-significant associations between social support and PTG [36, 37]. Given the limitations of cross-sectional studies, research has explored the longitudinal relationship. Some studies have shown that social support improves significantly after disasters and social support at Time 1 contributes to PTG at Time 2 [12, 38]. Although a temporal association between the two variables has already been examined, research has often overlooked the fact that PTG itself evolves over time, potentially influenced by baseline levels of social support.

### The relationship between short video exposure and PTSD symptoms and PTG: a trauma-related reminders perspective

In directly exposed survivors, disaster-related short videos primarily function as trauma-related reminders—salient visual/auditory cues tightly linked to the index trauma [39–42]. In the digital era, short videos, often shared through social media, act as channels for trauma-related reminders by repeatedly exposing individuals to distressing scenes, survivor testimonies, and real-time crisis updates. This type of exposure can heighten psychological distress, such as PTSD symptoms, among viewers, regardless of their direct involvement in the disaster. Furthermore, media exposure, while often distressing, can also serve as a coping mechanism for some individuals by providing information and creating a sense of social connectedness [43], which may foster PTG [44, 45].

Short video platforms have become increasingly popular in China in recent years, particularly during disasters [46]. During the 2021 Henan floods, a significant amount of short video content focused on real-time disaster updates, rescue operations, government responses, and personal experiences of those affected. These videos were shared through various channels, including public social media platforms such as WeChat Moments and Weibo, and private online chat. By providing firsthand visual and auditory representations of the disaster, short videos served as an immediate source of information but also acted as a form of trauma exposure, potentially intensifying emotional responses. Given the growing role of short video platforms in crisis communication, understanding their psychological impact on disaster victims is essential.

Research on the impact of disaster media coverage has documented a relationship between the amount of media exposure and psychological outcomes, particularly PTSD symptoms [39, 47]. As the trauma-related reminders, exposure to disaster-related media content can reactivate unpleasant memories of disasters and result in severe traumatization, which is also related to adverse mental health outcomes [48–50]. Cross-sectional studies [50, 51] and longitudinal studies [52, 53] have shown a consistent positive association between media exposure and subsequent PTSD symptoms. Nonetheless, a recent study focusing on posttraumatic stress symptoms trajectories identified three distinct categories, involving “resilience”, “recovery” and “deterioration” [54]. Moreover, higher levels of media use can predict a higher likelihood of being in the “recovery” class [54].

In addition to PTSD symptoms, PTG has become an increasingly important indicator in research on the association between media exposure and psychological outcomes. While the majority of studies have focused on the adverse effects of media exposure, Yu and his colleagues [55] reported that exposure to touching and encouraging content about the Sichuan earthquake via media coverage was correlated with high levels of PTG, the first finding of such relationships between media exposure and positive changes experienced by individuals. In addition, a study suggested that exposure to media coverage of the Great East Japan earthquake might promote PTG because it facilitated rumination [15]. Types of media use have also been examined in relation to PTG, with Raney et al. [56] finding that self-transcendent media use, which refers to media content that evokes feelings of awe, inspiration, or connectedness to something greater than oneself, mediated the relationship between hurricane-related stressors and PTG.

Taken together, the literature above provides empirical evidence for the positive influence of media exposure on disaster survivors’ posttraumatic recovery, but little is known about its effect on the trajectories of PTSD and PTG. Further, many forms of media exposure have been found to be associated with PTSD and PTG, including newspapers, broadcasting, television and internet [57]. However, with the advancement of media technology and the evolution of media ecology, there is still a lack of knowledge about the impact of short video exposure on mental health.

### The current study

This study attempts to investigate the predictive roles of social support and short video exposure on the initial levels and changes of PTSD symptoms and PTG by conducting a three-wave longitudinal study of victims of the 2021 Henan floods over a six-month period. To examine the relationships, we use the LGCM which can describe the influence of predictors on the initial levels (indicated by intercept) and changes (indicated by slope) of variables. The main research questions are as follows: (1) Does social support during the disaster predict the trajectories of PTSD symptoms and PTG during and after the disaster? More specifically, how does social support influence the initial levels and the changes of PTSD symptoms and PTG over time? (2) Does short video exposure during the disaster predict the trajectories of PTSD symptoms and PTG during and after the disaster? More specifically, how does short video exposure influence the initial levels and the changes of PTSD symptoms and PTG over time? Given that this is an exploratory study, we have no prior hypotheses.

## Method

### Participants and procedure

The current study collected data from July 20, 2021, to January 30, 2022. All participants were recruited via an online reward-based crowdsourcing platform, Credamo (https://www.credamo.com/), which is similar to Amazon’s “Mechanical Turk”.

The study assessed disaster victims at three time points: 0 (Wave 1), 3 (Wave 2), and 6 (Wave 3) months after the Henan floods. The first wave took place from July 20 to August 6, 2021, a period when the Henan citizens were still affected by the floods. Individuals with IP addresses from Henan province were invited to participate in the survey and 937 eventually completed the questionnaire. 224 participants were excluded because they failed to pass the attention check or reported “not applicable” to the flood exposure question. We included three attention check questions that required participants to choose a particular option prescribed by researchers. Participants were considered to be invalid if they did not answer all the questions correctly. Of the participants in the original sample, 713 valid participants were invited to participate in Wave 2 from October 30 to November 5, 2021, and 410 valid responses were collected. Wave 3 was conducted from January 28 to January 30, 2022, and 279 valid responses were collected. Ultimately, this study shows a tracking rate of 39.1%. The results of c² tests showed that the participants who dropped out of the study did not significantly differ from those who completed all waves in educational background (c² = 5.015, *p* =.414), marital status (c² = 7.656, *p* =.105) and annual family income (c² = 9.634, *p* =.210). However, drop-outs were significantly younger than completers and were more likely to be men (age: *W*_Mann−Whitney_ = 52020, *p* =.001; sex: c² = 8.538, *p* =.014). Additionally, we conducted analyses with Little’s Missing Completely at Random (MCAR) test to examine the missing data patterns among PTG and PTSD symptoms at three waves [58]. The null hypothesis of the MCAR test (the missing data were completely random) was not rejected (c² = 10.435, *p* =.107). The high missing rate can be attributed to the feature of online crowdsourcing platforms—their users have a high probability of dropping out [59]. For the current study, we only included the participants who finished all three study waves (*N* = 279).

We conducted a power analysis using the *semPower* package in R [60] to estimate the required sample size for our structural equation model. The analysis was based on an effect size of RMSEA = 0.08, a significance level of α = 0.05, and 25 degrees of freedom (as determined by the structure of our model). The results indicated that a minimum sample size of 144 was required. This suggests that our actual sample size was adequate given the complexity of the model.

### Measures

#### PTSD symptoms

PTSD symptom severity at each wave was assessed with the 17-item PTSD Checklist—Civilian Version (PCL-C; DSM-IV-concordant) [61]. We acknowledge that DSM-5- and ICD-11-aligned instruments (e.g., PCL-5, ITQ) are now the contemporary standards in trauma research. Although less commonly used today, the PCL-C still appears in recent studies [62–65]. To preserve comparability with the existing literature [66–68], we used the PCL-C to align with previous studies of disaster-related media exposure that employed this measure, facilitating cross-study comparison. Accordingly, we treat PCL-C scores as indices of DSM-IV-concordant PTSD symptom severity and do not make DSM-5/ICD-11 diagnostic inferences. While some reports suggest correspondence in overall severity and in associations with comorbidity between PCL-C and PCL-5 [69, 70], cross-measure equivalence is not assumed, and comparisons to PCL-5/ITQ studies should be made cautiously. The PCL-C includes 17 self-report items, and has shown good reliability among the Chinese population [16, 71, 72]. On a 5-point Likert scale ranging from 1 to 5 (1 = *not at all bothered*, 5 = *extremely severe*), participants were asked to rate the severity of these symptoms. Higher mean scores of all items indicated more severe PTSD symptoms. In the current study, Cronbach’s alphas for the PTSD scale are 0.956 at W1, 0.954 at W2 and 0.961 at W3, indicating excellent reliability.

#### PTG

The Posttraumatic Growth Inventory (PTGI) is a 21-item instrument for evaluating the levels of PTG [7]. The PTGI is the most widely used tool to assess the posttraumatic growth after traumatic events [54] and has been shown to have good internal consistency in China [73, 74]. Each of the 21 items was scored from 1 (*I did not experience this change*) to 6 (*I experienced this change to a great degree*), with higher mean scores of all items indicating higher levels of PTG. The Cronbach’s alpha of the PGTI in the current study are excellent (α = 0.970, 0.969, and 0.969 at W1, W2, and W3, respectively).

#### Social support

Social support in this study refers to individuals’ perceived level of social support during the disaster, measured using the C-SPS-10, the validated Chinese version of the 10-item Social Provisions Scale [71], which was originally developed by Caron [75] based on the work of Cutrona and Russell [76]. This scale assesses five dimensions: social integration, reassurance of worth, attachment, sense of reliable alliance, and guidance, with a total of 10 items. Each item was rated on a 4-point Likert scale (1 = *strongly disagree*, 4 = *strongly agree*), and higher mean scores of all items indicated more perceived social support during the disaster. The data of social support were collected in W1. The reliability of social support scale in the current study is good (Cronbach’s α = 0.813).

#### Short video exposure about the floods

Short video exposure about the floods, which has diverse scenarios, was measured by the scale implemented in a recent study [16]. The scale consists of 12 items (see Appendix), including 4 channel scenarios and 3 content scenarios, to operationalize the short video exposure during the Henan floods. The channel scenarios comprised short video exposure through WeChat moments, WeChat groups, WeChat personal chats and other social media platforms (including Weibo, Kwai, Bilibili, and TikTok). The content scenarios included exposure to short videos related to the situations in the disaster-affected areas, flood victims seeking for help, and help for flood victims. Participants reported the frequency of short video exposure in the 12 scenarios (4 channel scenarios × 3 content scenarios) on a 4-point Likert scale ranging from 1 (*not at all*) to 4 (*several times a day*). Higher mean scores of all items indicated more flood-related short video exposure. The data of short video exposure were collected in W1. The short video exposure scale exhibited good reliability in this study (Cronbach’s α = 0.873).

#### Control variables

Demographic data, including age (years), sex (male or female), educational background (below bachelor degree, bachelor degree, master degree and above), marital status (never married, others) and annual family income (below RMB 50,000, RMB 50,000–199,999, RMB 200,000 and above) were also collected.

### Statistical analysis

#### Descriptive analysis

Descriptive analysis was carried out to describe the basic characteristics of the participants. As the data for PTSD and PTG did not meet the ANOVA assumption of normal distributions, we used the nonparametric Friedman rank sum test to evaluate the changes in PTSD and PTG across three waves. *P*-values were also adjusted using the Bonferroni method.

#### Latent growth curve analysis

The following analyses were based on ratings of social support and short video exposure during the disaster, and ratings of PTSD symptoms and PTG at three time points (0-, 3-, and 6- months during and after the disaster). We applied the latent growth curve model (LGCM) to examine the effects of social support and short video exposure on intraindividual change of PTSD symptoms and PTG over six months.

**Fig. 1:**
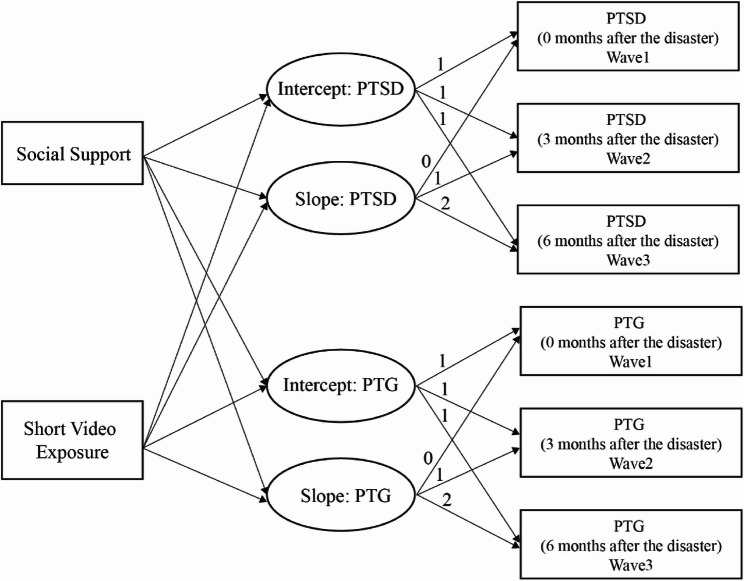
The latent growth curve model for PTSD and PTG. Intercept represents the baseline level (initial status) of PTSD/PTG symptoms immediately after the disaster (Wave 1), and slope represents the rate of change in PTSD/PTG symptoms across the three time points (0, 3, and 6 months after the disaster). Social support and short video exposure were modeled as predictors of both intercepts and slopes

To test these relationships, the analyses followed two stages. First, we determined the factor loadings of the paths and defined the model which is shown in Fig. 1. Factor loadings in the latent growth models represent the values of the time metric [53]. We constrained the paths from intercept factors to the observed variables of PTSD symptoms and PTG as 1, suggesting that the initial levels would not change for all participants across the three measures. The remaining factor loadings for the slope factors were set to 0, 1, and 2, indicating linear changes and the same time intervals (0-, 3-, and 6- months during and after the disaster). The final model used social support and short video exposure as predictors, age, sex, educational background, marital status and annual family income as control variables, and the parameters (intercept and slope) of PTSD symptoms and PTG as outcomes. Second, we tested the model using the R package *lavaan* [77]. We evaluated following model fit indices: chi-square, comparative fit index (CFI), tucker-lewis index (TLI), root mean square error of approximation (RMSEA), and standardized root mean square residual (SRMR).

The data and code that support the findings in both descriptive analysis and latent growth curve analysis of this work are presented on the Open Science Framework Platform (https://osf.io/hmn46).

## Results

### Descriptive results

Descriptive statistics for all variables are presented in Table 1. Among 279 participants, 158 (56.6%) were females and 121 (43.4%) were males. The mean age of the participants was 28.175 years. Only 19.7% of participants reported having below bachelor’s degree, 71.3% reported having a bachelor degree and 9.0% reported having a master degree and above. Over half of the participants (52.7%) were never married. Approximately 59.1% of participants reported a family income ranging from RMB 50,000 to 199,999, while 14.0% reported an income of RMB 200,000 and above, with the remainder (26.9%) reporting less than RMB 50,000.

The results of Friedman rank sum test showed that significant differences existed in PTSD symptoms across three waves (*p* <.001). Post hoc analysis revealed a statistically significant difference in the median of PTSD symptoms between Wave 1 and Wave 2 (*p* =.008), with median decreasing from 1.875 during the disaster to 1.778 three months following the disaster. There were no significant differences in PTG levels across three waves (*p* =.373).

**Table 1:** Descriptive statistics (*N* = 279)

Variables	*N* (%)	Mean (SD)	Min	Max
Age (years)	–	28.175 (8.025)	18.467	60.309
Sex (1 = female)	158 (56.6)	–	–	–
Education (ref.=below bachelor degree)				
Bachelor degree	199 (71.3)	–	–	–
Master degree and above	25 (9.0)	–	–	–
Marital status (1 = never married)	147 (52.7)	–	–	–
Annual family income (ref.= below RMB 50,000)				
RMB 50,000–199,999	165 (59.1)	–	–	–
RMB 200,000 and above	39 (14.0)	–	–	–
Social support	–	3.276 (0.362)	2.100	4.000
Short video exposure	–	3.074 (0.519)	1.667	4.000
PTSD W1	–	2.084 (0.837)	1.000	4.750
PTSD W2	–	1.962 (0.741)	1.000	4.500
PTSD W3	–	2.072 (0.848)	1.000	4.412
PTG W1	–	4.071 (1.176)	1.190	5.810
PTG W2	–	4.168 (1.094)	1.095	6.000
PTG W3	–	4.127 (1.069)	1.000	6.000

### The predictive role of social support on the trajectories of PTSD and PTG

Conditional LGCM using a linear model was applied to examine the predictive roles of social support and short video exposure on the trajectories of PTSD symptoms and PTG. The model showed a good fit to the data in general according to the statistics: c² = 55.789, *p* <.001, CFI = 0.974, TLI = 0.928, RMSEA = 0.066, SRMR = 0.030.

Our first research question was to investigate whether social support had an effect on the trajectories of PTSD symptoms and PTG. As is shown in Table 2, after controlling for demographic variables, social support during the disaster had no association with the trajectories of PTSD symptoms. Social support during the disaster had no statistically significant effects on the initial levels (*b* = 0.028, *p* =.857) nor changes (*b* = −0.061, *p* =.332) of PTSD symptoms over a six-month period. However, the impact of social support on the intercept (*b* = 1.285, *p* <.001) and slope (*b* = −0.173, *p* =.024) of PTG was significant. Those who had greater social support resources during the disaster were likely to have higher initial levels of PTG and lower rates of change in PTG over six months.

**Fig. 2:**
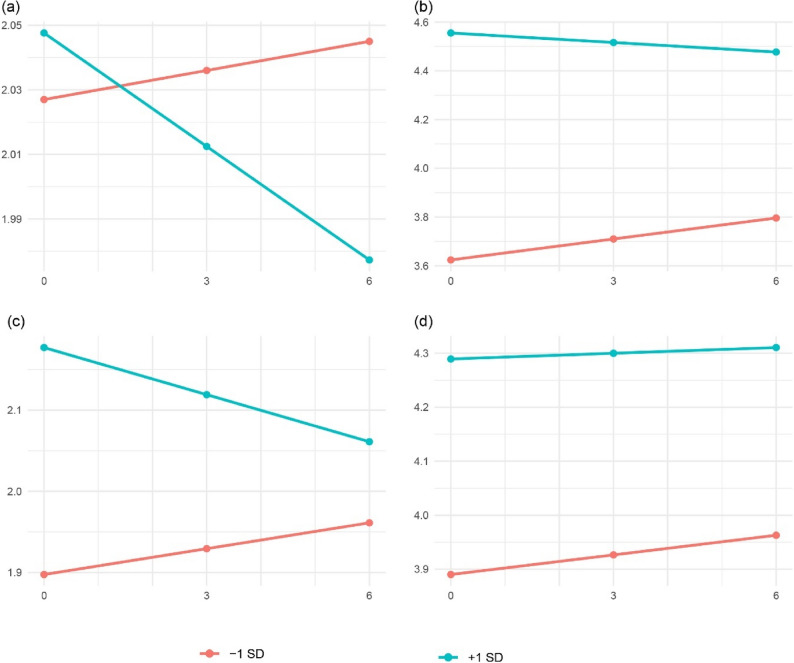
Model-implied trajectories from the LGCM. Lines show model-implied means, other covariates held at sample means. Time coded as 0, 3, and 6 months. **a** PTSD trajectories by baseline social support (±1 SD). **b** PTG trajectories by baseline social support (±1 SD). **c** PTSD trajectories by short-video exposure (±1 SD). **d** PTG trajectories by short-video exposure (±1 SD)

As shown in Fig. 2a and b, the model-implied trajectories for ± 1 SD largely overlap, although the lines suggest a directional pattern (higher support corresponding to slightly lower PTSD), the apparent separation is small, and we do not draw additional inferential conclusions from this visualization. By contrast, social support showed a dual association with PTG: participants at + 1 SD reported a higher PTG intercept (baseline), whereas their PTG slope was less positive (i.e., smaller increases over time) relative to − 1 SD. This pattern aligns with our framework that structural resources raise the starting point of adaptation but do not necessarily accelerate subsequent growth.

### The predictive role of short video exposure on the trajectories of PTSD and PTG

The second research question focused on whether short video exposure influenced the trajectories of PTSD symptoms and PTG. Table 2 shows that the effect of short video exposure on PTSD symptoms was significant, suggesting that short video exposure during the disaster predicted higher initial levels of PTSD symptoms (*b* = 0.269, *p* =.003) and negatively predicted the changes of PTSD symptoms (*b* = −0.086, *p* =.038). Individuals with more exposure to short videos during the disaster had more severe PTSD symptoms at baseline, with lower rates of change in PTSD symptoms over time. When examining the influence of short video exposure on the PTG, we found that short video exposure during the disaster significantly predicted the intercept of PTG positively (*b* = 0.384, *p* =.002), indicating that more frequent short video exposure during the disaster predicted higher levels of PTG at baseline. However, short video exposure did not significantly affect changes in PTG across three waves (*b* = −0.025, *p* =.633).

As depicted in Fig. 2c and d, the model-implied trajectories for ± 1 SD show clear separation at baseline and a flatter downward trend at + 1 SD, consistent with a trauma-reminder account. For PTG, exposure was positively associated with the intercept but not significantly associated with the slope; the ± 1 SD lines are largely parallel, indicating a higher baseline level of perceived growth at + 1 SD without evidence of faster change over time, and we do not draw additional inferential conclusions from this visualization.

### The predictive role of demographic and socioeconomic status

Marital status and family income showed unique associations with PTG only. Compared with other marital statuses, never-married participants reported a lower PTG intercept but a more positive PTG slope (i.e., faster increase over time). Participants with annual family income below RMB 50,000 also reported a lower PTG intercept. For PTSD, none of the demographic or socioeconomic covariates—including marital status and family income—were significantly associated with either the intercept or the slope. All other covariates in the model likewise showed no significant associations with the intercepts or slopes of either PTG or PTSD.

**Table 2:** Social support and short video exposure as predictors of latent growth curve model of PTSD and PTG

	PTSD intercept		PTSD slope		PTG intercept		PTG slope	
Variables	*b* (*SE*)	*p*	*b* (*SE*)	*p*	*b* (*SE*)	*p*	*b* (*SE*)	*p*
Social support	0.028 (0.158)	0.857	−0.061 (0.063)	0.332	1.285 (0.196)	0.000***	−0.173 (0.076)	0.024*
Short video exposure	0.269 (0.092)	0.003**	−0.086 (0.042)	0.038*	0.384 (0.122)	0.002**	−0.025 (0.052)	0.633
Age	0.001 (0.007)	0.911	−0.003 (0.004)	0.467	−0.003 (0.009)	0.733	0.004 (0.004)	0.337
Sex (1 = female)	−0.030 (0.097)	0.756	−0.022 (0.043)	0.607	−0.314 (0.106)	0.003**	0.004 (0.048)	0.932
Education (ref.=below bachelor degree)								
Bachelor degree	−0.125 (0.140)	0.373	0.054 (0.064)	0.404	0.075 (0.138)	0.590	−0.085 (0.064)	0.185
Master degree and above	−0.312 (0.194)	0.108	0.056 (0.081)	0.485	−0.415 (0.264)	0.115	−0.021 (0.100)	0.834
Marital status (1 = never married)	0.039 (0.126)	0.759	−0.066 (0.064)	0.305	−0.342 (0.146)	0.019*	0.107 (0.059)	0.070**
Annual family income (ref.= below RMB 50,000)								
RMB 50,000–199,999	0.003 (0.114)	0.976	0.074 (0.062)	0.231	0.351 (0.146)	0.016*	−0.021 (0.063)	0.743
RMB 200,000 and above	0.253 (0.179)	0.158	−0.036 (0.079)	0.645	0.448 (0.195)	0.021*	0.070 (0.073)	0.339

* *p* <.05. ** *p* <.01. *** *p* <.001

## Discussion

Guided by the social causation and stress-buffering perspectives—and conceptualizing short video exposure as media-based trauma-related reminders—this study examined whether social support during the disaster and short video exposure predicted trajectories of PTSD symptoms and PTG over the six months following the 2021 Henan floods. The results show the significance of social support resources and short video exposure in individuals’ psychiatric reactions during and after the disaster. By modeling longitudinal change rather than static outcomes and by incorporating a contemporary media form, this work adds to limited evidence on how evolving social resources and disaster-related media exposure shape PTSD and PTG over time.

Our primary question was how social support during the disaster predicted the intercepts and slopes of PTSD symptoms and PTG. Guided by the social causation and stress-buffering perspectives [20, 21], social support is considered a vital resource that can buffer the adverse effects of traumatic events and facilitate psychological recovery [19, 32]. Individuals with adequate support may redefine traumatic events and the stress reaction can be alleviated or eliminated by the intervention of social support [20]. Prior works often find that higher social support is associated with lower PTSD distress [78]. However, in our data, support measured during the disaster did not predict PTSD intercepts or slopes over six months. Specifically, social support during the disaster showed no significant association with either the PTSD intercept or PTSD slope across the six-month follow-up. Previous studies that have identified social support as a protective factor against PTSD symptoms have generally focused on the effect of pre- or post-disaster social support on posttraumatic distress, ignoring social support during disasters. For example, Chan et al. [79] suggested that perceived social support prior to Hurricane Katrina predicted the symptoms of posttraumatic stress. Another study found that belonging support of returning veterans, which was evaluated two weeks to 10 months after deployment, predicted decreases in PTSD symptom severity over time [80]. One plausible explanation is timing [43, 81–83], buffering effects may emerge predominantly after the acute phase, whereas our baseline “in-disaster” measure captures needs and actions under immediate threat rather than resources that shape subsequent symptom change. A second explanation is support-need mismatch: buffering benefits depend on a match between the type and timing of support and survivors’ needs. During the disaster, survivors may prioritize tangible, instrumental aid (e.g., food, shelter, medical care), whereas the C-SPS-10 primarily indexes emotional and relational support [71], potentially weakening its association with PTSD in this window. Relatedly, research on psychological debriefing shows mixed or adverse effects [84], and leading guidelines advise against routine single-session debriefing in the acute stage [85, 86]. This underscores that acute-phase support, when not matched to need or sustained over time, may not translate into reduced PTSD, highlighting the importance of type, timing, and continuity of resources in recovery.

Besides, social support during the disaster had a double impact on PTG. Firstly, it was found that social support had a positive association with the initial levels of PTG among participants. This result resonates with previous research [38], which has illustrated that social support leads to positive psychological adjustment (including PTG). Consistent with a social causation view, greater support indexes better access to informational, emotional, and instrumental resources, which can raise the baseline of growth [30]. Second, in-disaster support was negatively associated with the PTG slope over the six-month period. That is, higher support during the disaster was linked to a less positive PTG slope (i.e., smaller increases over time). This pattern differs from many post-event or cross-sectional studies that typically report more positive associations. One possible explanation relates to the temporal mismatch between support received during the disaster and survivors’ evolving needs in the recovery phase. Indeed, social support often declines and becomes unstable after the acute phase [71], which can constrain continued psychological growth. Thus, adequate early support may not translate into further gains if sustained support is lacking during recovery. Additionally, a ceiling effect may be present—individuals with already high levels of PTG at baseline may have less room for growth, resulting in a smaller change over time.

In addition to the relationships between social support and PTSD symptoms and PTG, we also aimed to identify the impact of short video exposure on the initial levels and changes of PTSD symptoms and PTG. The results of this study showed that participants with more frequent short video exposure reported higher levels of PTSD symptoms at initial and reported lower rates of change in PTSD symptoms over time. We consider these two findings to be particularly important for several reasons. Media exposure to traumatic events is verified as a risk factor for psychological disorders such as anxiety, peritraumatic distress, and PTSD symptoms [87]. But our longitudinal design adds evidence that short video exposure during a disaster may be associated with both elevated distress at baseline and a slower rate of psychological recovery over time. This finding provides a more dynamic understanding of how media exposure can impact mental health beyond the immediate aftermath.

Another notable finding was that more short video exposure during the disaster was positively associated with the PTG intercept but unrelated to the PTG slope. Within our theoretical framework, disaster-related short videos are conceptualized as media-based trauma-related reminders [39, 41], some exposure may co-occur with efficacy- or solidarity-oriented information that is linked to a higher baseline of perceived growth. At the same time, the absence of a slope effect indicates that exposure did not accelerate growth over time, consistent with a stress-buffering view in which sustained and well-matched supports, rather than acute exposure itself, shape the rate of increase in PTG. Because our measures did not differentiate content features (e.g., distressing imagery, imagery intensity, or exposure frequency), this association should be interpreted cautiously; future work should parse content, timing, and resource continuity to test these pathways more directly.

Moreover, consistent with our theoretical framework, lower baseline PTG among never-married and lower-income survivors likely reflects structural constraints on early access to informational, emotional, and instrumental resources immediately after the flood—especially when media-based trauma reminders heighten cognitive and emotional load [16, 39, 41]. By contrast, the more positive PTG slope observed for never-married participants is consistent with a stress-buffering account: although the absence of spousal support may dampen early benefit-finding, subsequent engagement with alternative buffers (e.g., community and peer networks, organized aid, efficacy-enhancing content) can facilitate catch-up growth. Income, primarily indexing material resources, aligns with a lower PTG intercept without clear slope differences in our data. Together, these patterns suggest that structural resources shape the starting point, whereas evolving social buffers shape the rate of increase in PTG over time.

This study suggests several implications for disaster victims’ psychological assistance during and after disasters. First, in the acute phase, the primary priority is to secure essential needs—safe shelter, potable water and sanitation, food, and basic medical care—consistent with need-hierarchy perspectives [88]. Psychosocial support should then be phased and integrated into medium- and long-term recovery: early efforts emphasize safety, practical assistance, and clear, reliable information; subsequent stages incorporate evidence-informed, scalable services (e.g., stepped care, targeted screening and referral, community-based supports). Strengthening structural resources can elevate the baseline of adaptation, whereas sustained, well-matched supports shape the rate of improvement over time. We do not infer that immediate psychosocial interventions uniformly prevent PTSD [84–86]; rather, we highlight the importance of timing, content, and continuity in designing and evaluating supports. Second, within the current media ecology, government agencies widely use social media for risk communication [89], short video platforms can serve as rapid channels for disseminating official risk and service information during disasters. In practice, governments may prioritize timely, specific, and verifiable guidance (e.g., what to do, and where/how to obtain services) and ensure accessibility (e.g., clear signposting, multilingual formats), while limiting highly graphic depictions that may act as trauma-related reminders, using content warnings options, and monitoring exposure frequency for unintended distress. Regarding algorithmic recommendation, the emphasis should be on accurate targeting with safeguards (e.g., authoritative-source whitelists, rate-limiting repetitive distressing content, and equity/misinformation audits). Because our measures did not differentiate content features, the present study cannot identify which specific forms of short video exposure are beneficial; therefore, any operational use should be principle-based and evaluative rather than prescriptive. Any putative effects on risk perceptions or recovery should be treated as hypotheses and subjected to future content-specific evaluations (e.g., parsing content type, timing, and exposure patterns).

Our findings point to several concrete directions for future research. First, given the limitations of cross-sectional designs in disaster psychology, future studies should incorporate longitudinal person-centered approaches, such as latent class growth analysis (LCGA) or growth mixture modeling (GMM), to capture distinct subgroups of symptom trajectories over time. This would provide a more nuanced understanding of heterogeneity in recovery patterns. Second, although this study did not assess cognitive reappraisal, future work should consider it as a potential mediator or moderator in the relationship between social resources and post-disaster outcomes. Empirical testing could involve using established instruments such as the Cognitive Emotion Regulation Questionnaire (CERQ) [90] or Emotion Regulation Questionnaire (ERQ) [91] to examine its interplay with variables like social support or media exposure. Third, our measure of short video exposure was developed for this study and has not been formally validated. To ensure psychometric soundness, future research should aim to develop and validate standardized instruments for assessing disaster-related media exposure, with attention to exposure frequency, emotional tone, and content type. Fourth, although this study employed a three-wave longitudinal design, the change in PTSD and PTG across time was relatively limited. The low variance in slopes may have constrained the ability to detect associations between predictors and growth trajectories. Additionally, potential floor or ceiling effects in the baseline distributions of PTSD and PTG could have further limited the detectable range of change. Future research may benefit from longer follow-up periods and the inclusion of more dynamic indicators of psychological adaptation.

Several limitations of this study should be acknowledged. First, one limitation of the present study is the relatively high attrition rate across waves. Although this is a common challenge in online crowdsourced studies [59], it may have affected the representativeness of the retained sample and introduced potential bias. Second, PTSD symptoms were measured using the PCL-C (DSM-IV-concordant) rather than PCL-5 (DSM-5) or ITQ (ICD-11), which are now more widely adopted. We chose the PCL-C to maintain comparability with prior literature on disaster-related media exposure, but this entails a construct-alignment trade-off: DSM-5/ICD-11 redefine the PTSD symptom structure (e.g., reconfigured clusters and additional symptoms) [69, 92], so the PCL-C may under- or over-represent domains emphasized in newer frameworks. Our trajectories should therefore be interpreted as PCL-C/DSM-IV-metric severity trajectories, not DSM-5/ICD-11 diagnoses, and cross-framework comparisons (e.g., to PCL-5/ITQ studies) may be non-equivalent despite strong internal consistency here. This measurement choice may influence intercepts and slopes if DSM-5/ICD-11-salient domains (e.g., negative cognitions/mood, specific arousal features) are not fully captured by the PCL-C. To mitigate over-interpretation, we avoid diagnostic claims and cluster-specific inferences and frame results as severity trajectories. Future research in similar contexts should adopt PCL-5 or ITQ, consider functional-impairment criteria where appropriate, and, in longitudinal designs, evaluate measurement invariance both longitudinally (across waves) and across key subgroups (e.g., sex, age, marital status, income, exposure severity) to ensure that differences in intercepts and slopes reflect true change rather than shifts in measurement properties [93–95], when bridging instruments, use cross-walk approach to align metrics across tools [96]. Third, only the common protective or risk factors influencing PTSD symptoms and PTG trajectories were tested in the current study. More factors that may influence PTSD symptoms and PTG trajectories in the affected population should be included. Fourth, we only measured predictive variables (social support and short video exposure) at Wave 1, but it is possible that they will change over time. Future studies can track their variations with the development of PTSD symptoms and PTG. Fifth, the changes in PTSD symptoms and PTG over time appeared to show quadratic trends, which can be examined with at least four waves of data. The current study only examined linear changes due to the limitation of the three-wave data. Future studies can collect four-wave data to examine the potential non-linear trends in the similar research contexts. Finally, the short video exposure was measured via the frequency of 12 short video exposure scenarios over the past two weeks, which may have limited generalization of adopting them in further studies’ scenarios.

## Data Availability

The data and code that support the findings in both descriptive analysis and latent growth curve analysis of this work are presented on the Open Science Framework Platform (https://osf.io/hmn46).

## Appendix

Short Video Exposure About the Floods Questionnaire

Instruction:

In the past two weeks, have you watched the following types of short videos to learn about the situation in disaster-affected areas of Henan?

For each item below, please indicate the frequency of exposure using the following scale:

- 1 = Not at all
- 2 = A few days a week
- 3 = Almost every day
- 4 = Several times a day

### Section 1: Videos about disaster-stricken areas and victims’ situations

Videos shared by others in WeChat Moments

Videos shared in WeChat groups

Videos received through private chats with WeChat friends

Videos on platforms such as Weibo, Kwai, Bilibili, or TikTok

### Section 2: Videos about people seeking help in disaster-stricken areas

Videos shared by others in WeChat Moments

Videos shared in WeChat groups

Videos received through private chats with WeChat friends

Videos on platforms such as Weibo, Kwai, Bilibili, or TikTok

### Section 3: Videos about support and assistance provided to disaster-stricken areas

Videos shared by others in WeChat Moments

Videos shared in WeChat groups

Videos received through private chats with WeChat friends

Videos on platforms such as Weibo, Kwai, Bilibili, or TikTok

## Abbreviations

CFI: Comparative Fit Index
C-SPS-10: Chinese Social Provisions Scale–10 Item Version
DSM-IV: Diagnostic and Statistical Manual of Mental Disorders, Fourth Edition
ICD-11: International Classification of Diseases 11th Revision
ITQ: International Trauma Questionnaire
LGCM: Latent Growth Curve Model
MCAR: Missing Completely at Random
PCL-C: PTSD Checklist–Civilian Version
PCL-5: PTSD Checklist for DSM-5
PTG: Posttraumatic Growth
PTGI: Posttraumatic Growth Inventory
PTSD: Posttraumatic Stress Disorder
RMSEA: Root Mean Square Error of Approximation
SEM: Structural Equation Modeling
SRMR: Standardized Root Mean Square Residual
TLI: Tucker–Lewis Index
